# How Molecular Chiralities of Bis(mandelato)borate Anions Affect Their Binding Structures With Alkali Metal Ions and Microstructural Properties in Tetraalkylphosphonium Ionic Liquids

**DOI:** 10.3389/fchem.2020.00065

**Published:** 2020-02-12

**Authors:** Han-Wen Pei, Bin Li, Aatto Laaksonen, Yong-Lei Wang

**Affiliations:** ^1^Arrhenius Laboratory, Department of Materials and Environmental Chemistry, Stockholm University, Stockholm, Sweden; ^2^School of Chemical Engineering and Technology, Sun Yat-sen University, Zhuhai, China; ^3^State Key Laboratory of Materials-Oriented and Chemical Engineering, Nanjing Tech University, Nanjing, China; ^4^Centre of Advanced Research in Bionanoconjugates and Biopolymers, Petru Poni Institute of Macromolecular Chemistry, Iasi, Romania

**Keywords:** molecular chiralities, bis(mandelato)borate anions, DFT calculations and atomistic simulations, tetraalkylphosphonium ionic liquids, alkali metal ions

## Abstract

Spiroborate anion-based inorganic electrolytes and ionic liquids (ILs) have fascinating electrochemical and tribological properties and have received widespread attention in industrial applications. The molecular chiralities of spiroborate anions have a significant effect on the microstructures and macroscopic functionalities of these ionic materials in application and thus deserve fundamental consideration. In the current work, we performed quantum chemistry calculations to address the binding strength and coordination structures of chiral bis(mandelato)borate ([BMB]) anions with representative alkali metal ions, as well as the electronic properties of alkali metal ion-[BMB] ion pair complexes. The optimized [BMB] conformers are categorized into V-shaped, bent, and twisted structures with varied electrostatic potential contours and conformational energies and distinct alkali metal ion-[BMB] binding structures. Alkali metal ions have additional associations with phenyl groups in V-shaped [BMB] conformers owing to preferential cation-π interactions. Furthermore, the effects of the molecular chiralities of [BMB] anions on the thermodynamics and microstructural properties of tetraalkylphosphonium [BMB] ILs were studied by performing extensive atomistic interactions. Oxygen atoms in [BMB] anions have competitive hydrogen bonding interactions with hydrogen atoms in cations depending on the molecular chiralities and steric hindrance effects of [BMB] anions. However, the molecular chiralities of [BMB] anions have a negligible effect on the liquid densities of tetraalkylphosphonium [BMB] ILs and the spatial distributions of boron atoms in anions around phosphorous atoms in cations. Enlarging tetraalkylphosphonium cation sizes leads to enhanced cation-anion intermolecular hydrogen bonding and Coulombic interactions due to enhanced segregation of polar groups in apolar networks in heterogeneous IL matrices, as verified by scattering structural functions.

## 1. Introduction

Ionic liquids (ILs) represent an intriguing category of molten salts solely composed of inorganic or organic anions and, most commonly, organic cations with melting points at or close to room temperature (Castner et al., 2011; Hayes et al., 2015; Zhang et al., 2016; Bedrov et al., 2019). In recent years, ILs have received significant attention in diverse academic and industrial communities due to their multifaceted physicochemical properties, such as non-flammability, negligible volatility, reasonable viscosity-temperature features, high thermal-oxidative stabilities, wide electrochemical windows, and outstanding affinities to polar and apolar compounds (Armand et al., 2009; Zhou et al., 2009; Castner et al., 2011; Wang et al., 2012; Hayes et al., 2015; Dai et al., 2017; Bedrov et al., 2019). These fascinating characteristics can be widely tuned in a controllable fashion via a judicious selection of ion moieties and by mutating specific atoms in constituent ion species (Kashyap et al., 2013; Wang et al., 2014, 2017b; Jankowski et al., 2016; Wu et al., 2016), making ILs exceptionally attractive and reliable alternatives to conventional molecular liquids in applications spanning from solvents in materials synthesis to electrolytes in electrochemical devices (Armand et al., 2009; Zhou et al., 2009; Castner et al., 2011; Abbott et al., 2013; Zhang et al., 2016; Dai et al., 2017; Watanabe et al., 2017; Bedrov et al., 2019).

ILs consisting of spiroborate anions coupled with tetraalkylphosphonium, pyrrolidinium, and imidazolium cations present practical tribological advantages and can be used as alternative high-performance lubricants and lubricant additives in tribology due to their outstanding friction-reducing and anti-wear performance in comparison with conventional fully formulated engine oil in tribological contact with a wide variety of solid materials (Shah et al., 2011, 2013, 2018; Taher et al., 2014; Gusain et al., 2017; An et al., 2018; Pilkington et al., 2018; Hjalmarsson et al., 2019; Rohlmann et al., 2019). Therefore, the number of fundamental studies and industrial applications of spiroborate anion-based ILs has been growing rapidly in recent years.

Besides variations in cation structures, the molecular chiralities of spiroborate anions have a significant effect on the microstructures and dynamics, mesoscopic liquid morphologies, and macroscopic functionalities of these ILs in tribology, electrochemistry, and pharmaceutical chemistry (Yu et al., 2008; Absalan et al., 2012; Sedghamiz and Bahrami, 2019). The bis(mandelato)borate ([BMB]) anion-based ILs are used as selectors for chiral discrimination of propranolol enantiomers (Sedghamiz et al., 2018; Sedghamiz and Bahrami, 2019). Both experimental (Absalan et al., 2012) and computational studies (Sedghamiz et al., 2018; Sedghamiz and Bahrami, 2019) showed that [BMB] anions contribute to distinct hydrogen bonding (HB) and π-π stacking interactions with propranolol enantiomers, leading to the formation of propranolol-[BMB] complexes with varied molecular stabilities depending on the specific chiralities of [BMB] anions. In another work, Wong and coworkers presented experimental evidence that [BMB] anions are effective resolving agents for the resolution of a chemically diverse range of racemic cations via metathesis crystallization (Wong et al., 2015, 2017, 2018).

**Figure 1:**
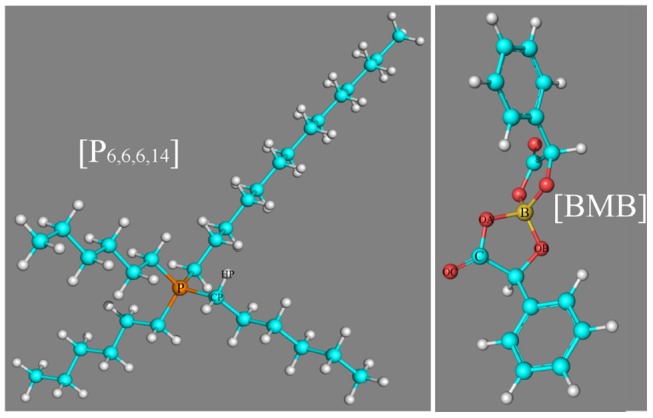
Molecular structures of the [P_6,6,6,14_] cation and B(S)-Man(SR) [BMB] conformer, as well as representative atom types in these two ion species.

The significance of [BMB] anions with different molecular chiralities being sensitive to local ionic environments motivates us to explore their delicate associations with alkali metal ions and tetraalkylphosphonium cations, which may provide valuable information for the selection and design of suitable electrolytes and lubricant additives with desirable physicochemical, structural, and functional properties for applications in electrochemistry and tribology (Shah et al., 2011, 2013, 2018; Jankowski et al., 2016; An et al., 2018; Pilkington et al., 2018; Franco et al., 2019; Hjalmarsson et al., 2019). In the current work, we performed intensive density functional theory (DFT) calculations to study the effects of the molecular chiralities of [BMB] anions on their specific binding structures with representative alkali metal ions and, thereafter, extensive atomistic simulations to explore the delicate interactions of [BMB] anions with tetraalkylphosphonium cations with varied alkyl substituents. Three tetraalkylphosphonium cations, namely tetrabutylphosphonium ([P_4,4,4,4_]), tributyloctylphosphonium ([P_4,4,4,8_]), and trihexyltetradecylphosphonium ([P_6,6,6,14_]), and six [BMB] conformers with different molecular chiralities are considered in present work. A schematic molecular structure of the [P_6,6,6,14_] cation and B(S)-Man(SR) [BMB] conformer, as well as representative atom types in these two ions are present in Figure 1.

## 2. Binding Structures of [BMB] Anions With Alkali Metal Ions

DFT calculations were first performed to optimize the molecular geometries of single [BMB] conformers and, thereafter, their tight binding ion-pair structures with alkali metal ions, respectively, using Gaussian 09 package (Frisch et al., 2009) (version D.01) at the B3LYP/6-311+G(d) level of theory (Lee et al., 1988) with Grimme's-D3 dispersion correction (Grimme et al., 2010). This dispersion correction is appropriate over medium (≈ 2–5 Å) and long ranges (> 5 Å) and is an effective method for obtaining the binding structures of complexes with reduced computational cost. Only molecular geometries with all positive vibrational frequencies were taken into consideration to ensure that the obtained anion structures of [BMB] conformers are in fact true minima in the corresponding energy landscapes.

**Figure 2:**
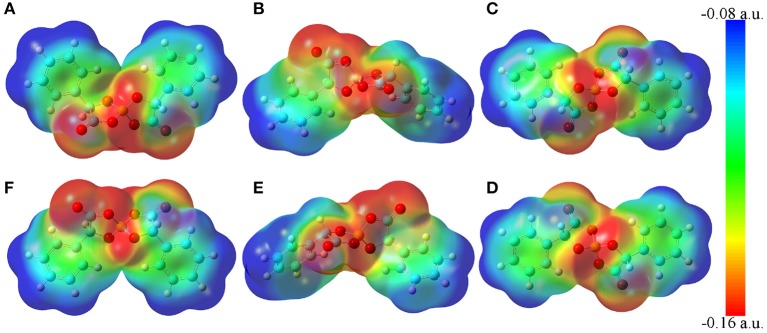
Molecular electrostatic potential contours of six [BMB] conformers with different molecular chiralities obtained from DFT calculations. **(A)** B(R)-Man(RR), **(B)** B(R)-Man(RS), **(C)** B(R)-Man(SS), **(D)** B(S)-Man(RR), **(E)** B(S)-Man(SR), and **(F)** B(S)-Man(SS).

The molecular electrostatic potential contours of six [BMB] conformers based on their respective optimized ion structures are present in Figure 2. These optimized [BMB] conformers have remarkable molecular shapes and can be categorized into three pairs, that is, the B(R)-Man(RR) and B(S)-Man(SS) [BMB] conformers (Figures 2A,F) are characterized by V-shaped structures, the B(R)-Man(RS) and B(S)-Man(SR) [BMB] conformers (Figures 2B,E) have bent conformations, and the B(R)-Man(SS) and B(S)-Man(RR) [BMB] conformers (Figures 2C,D) have twisted configurations. Each pair has symmetric ion structures and electrostatic potential contours and is characterized by similar relative conformational energies, dipole moments, and molecular volumes. These computational data are listed in Table 1. Previous DFT calculations indicated that boron-based chiral anions are energetically similar in diastereomeric [BMB] systems (Wong et al., 2018). In the current work, the B(R)-Man(RS) and B(S)-Man(SR) [BMB] conformers have the lowest conformational energies, whereas the B(R)-Man(SS) and B(S)-Man(RR) [BMB] conformers have the highest conformational energies, attributing to unbalanced intramolecular forces within [BMB] anionic frameworks. It is noted that the conformational energy difference between all these [BMB] conformers is less than 1 kJ/mol, indicating that these [BMB] conformers may co-exist in IL samples, which is consistent with the results obtained from NMR spectra (Wong et al., 2015). However, the precipitation of twisted B(R)-Man(SS) and B(S)-Man(RR) [BMB] conformers rather than the V-shaped B(R)-Man(RR) and B(S)-Man(SS) [BMB] conformers is always observed in crystallized salts (Wong et al., 2015, 2017). DFT calculations showed that, in all [BMB] conformers, four oxygen atoms exhibit tetrahedral-like distributions around central boron atoms, whereas two oxalato rings have constrained distributions. The addition of phenyl groups to central oxalato moieties results in distorted orientations of phenyl groups around oxalato rings. These distinct conformational variations of [BMB] anions lead to their varied dipole moments and molecular volumes, as listed in Table 1. The central moieties in [BMB] anions are polar and have a significant negative charge, leading to varied HB capabilities of three oxygen atom types with HP atoms (as labeled in Figure 1) in tetraalkylphosphonium cations (Wang et al., 2015a) and with polar solute molecules, like water (Wang et al., 2015a, 2016).

**Table 1:** Relative conformational energies (RCEs) (in kJ/mol), dipole moments (DMs) (in Debye), and molecular volumes (in cm^3^/mol) of single [BMB] conformers, and relative binding energies (RBEs) (in kJ/mol) of varied [BMB] conformers in coordinating alkali metal ions (Li^+^, Na^+^, and K^+^), as determined from DFT calculations.

	**Single [BMB] conformers**			**Alkali metal ion-[BMB] ion pairs**		
**Conformers**	**RCEs**	**DMs**	**Volumes**	**Li-BMB**	**Na-BMB**	**K-BMB**
B(R)-Man(RR)	0.0982	6.8945	191.770	−3.0179	−7.5136	−9.5608
B(R)-Man(RS)	0.0000	4.9898	225.871	−0.3833	−1.1197	−4.2263
B(R)-Man(SS)	0.4638	1.6472	248.534	−0.1394	−0.8400	0.0000
B(S)-Man(RR)	0.4642	1.6474	249.508	0.0000	0.0000	−0.3294
B(S)-Man(SR)	0.0020	4.9898	226.122	−1.9355	−0.9143	−4.6534
B(S)-Man(SS)	0.0986	6.8947	192.447	−3.0175	−7.5135	−8.9812

**Figure 3:**
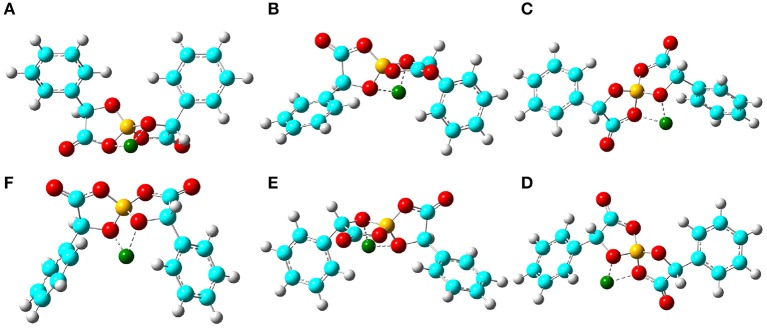
Optimized ion-pair structures of an Li^+^ ion in coordinating [BMB] anions with varied molecular chiralities determined from DFT calculations. The hydrogen, boron, carbon, and oxygen atoms in [BMB] anions are represented by white, yellow, cyan, and red spheres, respectively, and Li^+^ ions are represented by green spheres. **(A)** B(R)-Man(RR), **(B)** B(R)-Man(RS), **(C)** B(R)-Man(SS), **(D)** B(S)-Man(RR), **(E)** B(S)-Man(SR), and **(F)** B(S)-Man(SS).

**Figure 4:**
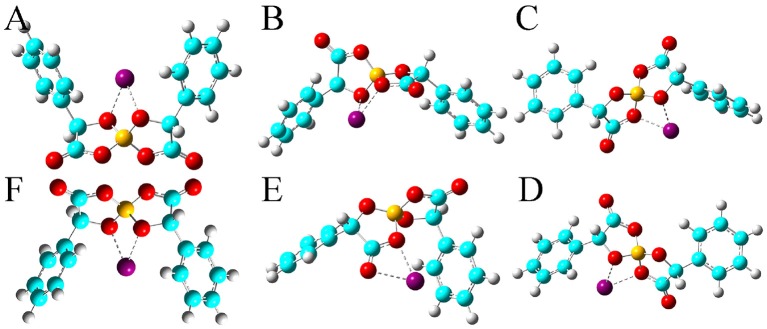
Optimized ion-pair structures of an Na^+^ ion in coordinating [BMB] anions with varied molecular chiralities determined from DFT calculations. The hydrogen, boron, carbon, and oxygen atoms in [BMB] anions are represented by white, yellow, cyan, and red spheres, respectively, and Na^+^ ions are represented by purple spheres. **(A)** B(R)-Man(RR), **(B)** B(R)-Man(RS), **(C)** B(R)-Man(SS), **(D)** B(S)-Man(RR), **(E)** B(S)-Man(SR), and **(F)** B(S)-Man(SS).

**Figure 5:**
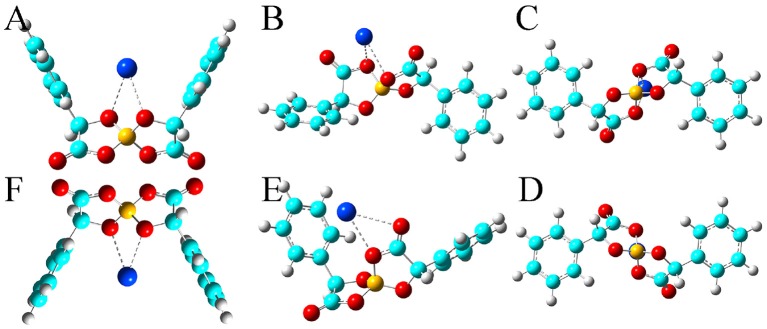
Optimized ion-pair structures of a K^+^ ion in coordinating [BMB] anions with varied molecular chiralities determined from DFT calculations. The hydrogen, boron, carbon, and oxygen atoms in [BMB] anions are represented by white, yellow, cyan, and red spheres, respectively, and K^+^ ions are represented by blue spheres. **(A)** B(R)-Man(RR), **(B)** B(R)-Man(RS), **(C)** B(R)-Man(SS), **(D)** B(S)-Man(RR), **(E)** B(S)-Man(SR), and **(F)** B(S)-Man(SS).

In addition, the optimized binding structures of varied [BMB] conformers with representative alkali metal ions (Li^+^, Na^+^, and K^+^) were determined from intensive DFT calculations at the same level of theory as that used for the calculation of single [BMB] conformers. Furthermore, the binding energies (in kJ/mol) of varied [BMB] conformers with alkali metal ions were determined as the energy difference between the optimized alkali metal ion-[BMB] complexes and the summation of the conformational energies of separately optimized ion species. These binding energies are corrected for basis sets superposition error (BSSE) using the counterpoise procedure (Boys and Bernardi, 1970). The relative binding energies of three alkali metal ions with six [BMB] conformers are listed in Table 1, and the corresponding optimized ion-pair structures are shown in Figures 3–5, respectively.

All these alkali metal ions exhibit specific associations with central polar moieties of [BMB] conformers via coordinating oxygen atoms in [BMB] anions. For a given alkali metal ion, both B(R)-Man(RR) and B(S)-Man(SS) [BMB] conformers have the most stable alkali metal ion-[BMB] binding structures, as verified from the relative binding energies shown in Table 1, due to the intrinsic V-shaped distributions of two phenyl groups in [BMB] anionic frameworks. All three alkali metal ions are intrinsically coordinated with B(R)-Man(RR) and B(S)-Man(SS) [BMB] conformers in local cavities of V-shaped conformations. For bent and twisted [BMB] conformers, alkali metal ions either coordinate with two oxygen atoms in central BO_4_ moieties or with one oxygen in BO_4_ moieties and the other one in C=O moieties depending on the delicate interactions of alkali metal ions with specific atoms in [BMB] conformers.

In addition, alkali metal ions have preferential associations with phenyl groups in V-shaped B(R)-Man(RR) and B(S)-Man(SS) [BMB] conformers. It is demonstrated that an increase of alkali metal ion sizes from Li^+^ to Na^+^ and K^+^ ions leads to their distinct cation-π coordinations with phenyl groups (Dougherty, 2012). Larger alkali metal ions have stronger cation-π interactions and therefore have larger binding energies with V-shaped B(R)-Man(RR) and B(S)-Man(SS) [BMB] conformers. This may lead to distinct applications of [BMB] anions in alkali metal ion batteries, in which alkali metal ion-[BMB] adducts are effective charge carriers contributing to significant ion conductivities in electrochemical devices.

**Figure 6:**
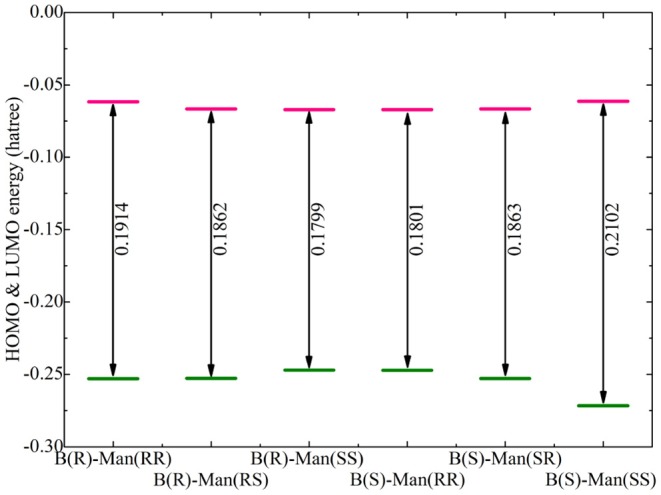
Computational HOMO and LUMO energies (in Hartree), and energy gaps (LUMO-HOMO) for Li[BMB] ion-pair complexes with anions of varied molecular chiralities. These computational results were obtained from DFT calculations at the B3LYP/6-311+G(d) level of theory with Grimme's-D3 dispersion correction.

Additional electronic properties of alkali metal ion-[BMB] complexes were calculated based on their optimized ion-pair structures. The energy levels of the highest occupied molecular orbital (HOMO) and the lowest unoccupied molecular orbital (LUMO) for the optimized Li[BMB] ion-pair structures are shown in Figure 6. According to the frontier molecular orbital theory, the HOMO and LUMO energies are useful for evaluating the electron-donating and -accepting abilities of ion species (Dong et al., 2012; Garćıa et al., 2015). Higher values of HOMO energy and lower values of LUMO energy indicate a larger tendency for ion species to donate and accept electrons, respectively. In addition, the HOMO and LUMO energies are directly related to ionization potential and electron affinity and give quantitative information on the possible electron transition between alkali metal ions and [BMB] anions. The energy difference between HOMO and LUMO is an important parameter determining the molecular-electrical transport properties, molecular stabilities, and liquid phase electrochemical windows of alkali metal ion-[BMB] complexes (Dong et al., 2012; Garćıa et al., 2015; Yllö and Zhang, 2019). It is shown that the HOMO and LUMO energy levels and energy gaps for the six Li[BMB] ion-pair complexes generally follow a similar tendency as the relative binding energies (listed in Table 1) for these [BMB] conformers in coordinating Li^+^ ions. The twisted B(R)-Man(SS) and B(S)-Man(RR) [BMB] conformers have the lowest energy gap among the three categories, indicating that Li^+^ ions tend to have higher reactivities with twisted [BMB] configurations than with other [BMB] conformers. The energy gaps for bent B(R)-Man(RS) and B(S)-Man(SR) [BMB] conformers are slightly larger than those for twisted [BMB] configurations. It is noteworthy that for the two V-shaped structures, the Li[BMB] (B(S)-Man(SS)) ion pair has a larger energy gap than does the Li[BMB] (B(R)-Man(RR)) ion pair. This indicates that the Li[BMB] (B(S)-Man(SS)) ion pair has higher stability and opposing charge transfer than other Li[BMB] ion-pair structures, since this ion-pair complex opposes changes in its electron density and distribution. In addition, the Li[BMB] (B(S)-Man(SS)) ion-pair complex has a lower HOMO energy than the Li[BMB] (B(R)-Man(RR)) ion-pair structure, indicating that the former complex has a distinct electrochemical stability and a larger electrochemical window than other Li[BMB] ion-pair structures when it is used as a solvent electrolyte in Li-ion batteries (Franco et al., 2019; Nilsson-Hallén et al., 2019).

**Figure 7:**
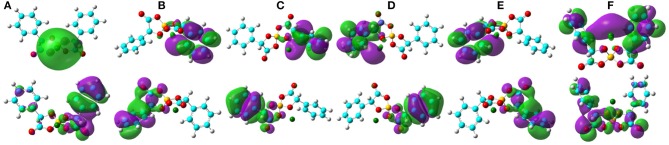
HOMO (lower panels) and LUMO (upper panels) contours for the optimized Li[BMB] ion-pair structures with anions of varied molecular chiralities. These orbital isosurfaces were drawn with a contour value of 0.02. The purple and green isosurfaces of HOMO and LUMO indicate negative and positive values, respectively. The hydrogen, boron, carbon, and oxygen atoms in [BMB] anions are represented by white, yellow, cyan, and red spheres, respectively, and Li^+^ ions are represented by green spheres. **(A)** B(R)-Man(RR), **(B)** B(R)-Man(RS), **(C)** B(R)-Man(SS), **(D)** B(S)-Man(RR), **(E)** B(S)-Man(SR), and **(F)** B(S)-Man(SS).

The computational HOMO and LUMO contours for the optimized Li[BMB] ion-pair structures are shown in Figure 7. It is clearly demonstrated that HOMO positions are mainly localized on phenyl groups and partially extended to oxalato ring structures for all Li[BMB] ion-pair complexes. This observation is attributed to the π-states of aromatic ring structures including both phenyl and oxalato ring moieties. However, the LUMO distributions are concentrated over Li^+^ ions for V-shaped B(S)-Man(SS) and B(R)-Man(RR) [BMB] conformers and are located primarily around Li^+^ ions and phenyl groups for twisted B(R)-Man(SS) and B(S)-Man(RR) [BMB] conformers as well as for bent B(R)-Man(RS) and B(S)-Man(SR) [BMB] conformers. The occurrence of electron transfer from HOMO to LUMO centers leads to an electron density transfer from one phenyl group to the other in the same anion framework via central oxalato moieties. Therefore, [BMB] anions are primarily responsible for the charge transfer process between Li^+^ ions and anions. Furthermore, we can assume that intramolecular charge transfer is enhanced within an anion framework for anions with larger ion hydrophobicity.

**Figure 8:**
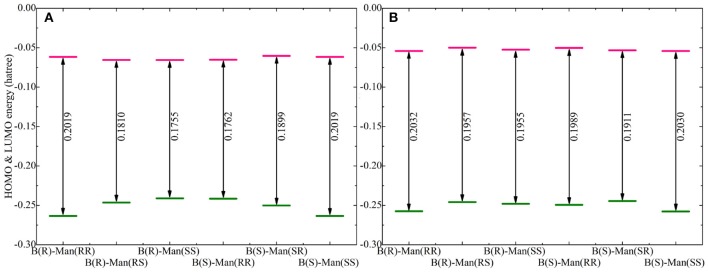
Computational HOMO and LUMO energies (in Hartree) and energy gaps (LUMO-HOMO) for **(A)** Na[BMB] and **(B)** K[BMB] ion-pair complexes with anions of varied molecular chiralities. These computational results were obtained from DFT calculations at the B3LYP/6-311+G(d) level of theory with Grimme's-D3 dispersion correction.

**Figure 9:**
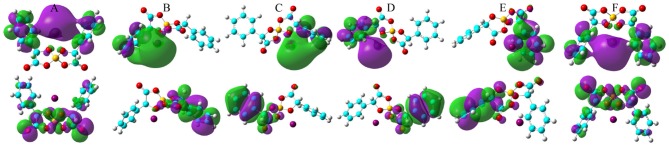
HOMO (lower panels) and LUMO (upper panels) contours for the optimized Na[BMB] ion-pair structures with anions of varied molecular chiralities. These orbital isosurfaces were drawn with a contour value of 0.02. The purple and green isosurfaces of HOMO and LUMO indicate negative and positive values, respectively. The hydrogen, boron, carbon, and oxygen atoms in [BMB] anions are represented by white, yellow, cyan, and red spheres, respectively, and Na^+^ ions are represented by purple spheres. **(A)** B(R)-Man(RR), **(B)** B(R)-Man(RS), **(C)** B(R)-Man(SS), **(D)** B(S)-Man(RR), **(E)** B(S)-Man(SR), and **(F)** B(S)-Man(SS).

**Figure 10:**
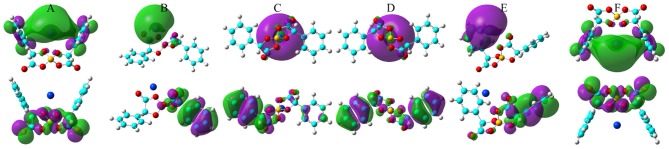
HOMO (lower panels) and LUMO (upper panels) contours for the optimized K[BMB] ion-pair structures with anions of varied molecular chiralities. These orbital isosurfaces were drawn with a contour value of 0.02. The purple and green isosurfaces of HOMO and LUMO indicate negative and positive values, respectively. The hydrogen, boron, carbon, and oxygen atoms in [BMB] anions are represented by white, yellow, cyan, and red spheres, respectively, and K^+^ ions are represented by blue spheres. **(A)** B(R)-Man(RR), **(B)** B(R)-Man(RS), **(C)** B(R)-Man(SS), **(D)** B(S)-Man(RR), **(E)** B(S)-Man(SR), and **(F)** B(S)-Man(SS).

The HOMO and LUMO energy levels for the optimized Na[BMB] and K[BMB] ion-pair structures are shown in Figure 8. These HOMO and LUMO energies and energy gaps exhibit similar features as those for Li[BMB] ion-pair structures, indicating that these alkali metal ions have similar coordination patterns with [BMB] anions but with different interaction strength. In addition, the HOMO and LUMO contours for the optimized Na[BMB] and K[BMB] ion-pair structures shown in Figures 9, 10 present a qualitative difference from those for the optimized Li[BMB] ion-pair complexes shown in Figure 7 owing to the different ion parameters of Li^+^, Na^+^, and K^+^ ions. Nevertheless, the distinct coordination patterns and interaction strengths of different [BMB] conformers with three alkali metal ions may lead to the varied stabilities and electrochemical windows of alkali metal ion-[BMB] complexes. These striking physicochemical properties may contribute to striking applications of [BMB] anions in alkali metal ion batteries, in which alkali metal ion-[BMB] complexes are effective charge carriers contributing to significant ion conductivities in electrochemical devices.

## 3. Atomistic Simulations of Tetraalkylphosphonium [BMB] Ionic Liquids

### 3.1. Atomistic Simulation Methodology

The atomistic force field parameters of tetraalkylphosphonium cations and [BMB] anions are taken from a force field systematically developed in our previous studies based on the AMBER framework (Wang et al., 2014, 2015b). The cross-interaction parameters between different atom types are obtained from the Lorentz-Berthelot combination rules. It is noteworthy that during force field development for tetraalkylphosphonium orthoborate ILs in our previous work (Wang et al., 2014), both tetraalkylphosphonium cations and orthoborate anions were described by unity charges and all atomic partial charges were determined by fitting the molecular electrostatic potential generated from DFT calculations of individual ions. In recent computational studies, it has been suggested that a down-scaling of atomic partial charges is an effective way to account for polarization and charge transfer effects between ion species and thereby can improve the reliability of calculations of the dynamical and transport quantities of ILs (Morrow and Maginn, 2002; Ishizuka and Matubayasi, 2016). It was identified in a recent study that a charge scaling factor of 0.8 could provide liquid viscosities for [P_6,6,6,14_][BMB] and [P_6,6,6,14_]Cl ILs that were as reliable as those obtained from experimental measurements within a wide temperature interval of 373-463 K (Sarman et al., 2018). Therefore, in the current work, all atomic partial charges for atoms in tetraalkylphosphonium cations and [BMB] anions are uniformly rescaled using a scaling factor of 0.8. It should be noted that even though the adopted charge scaling factor of 0.8 is an empirical correction of electrostatic polarization and charge transfer effects between tetraalkylphosphonium cations and [BMB] anions, it gives good performance in describing the thermodynamics and microstructural quantities of tetraalkylphosphonium [BMB] ILs.

In present atomistic simulations, each simulation system consists of a varied number of tetraalkylphosphonium [BMB] ion pairs, having approximately 35,000 atoms. More specifically, [P_4,4,4,4_][BMB], [P_4,4,4,8_][BMB], and [P_6,6,6,14_][BMB] IL systems are composed of 400, 352, and 259 ion pairs, respectively. Atomistic simulations were performed using the GROMACS 5.0.7 package (Abraham et al., 2015) with cubic periodic boundary conditions. The equations of motion were integrated using a classical velocity Verlet leapfrog integration algorithm with a time step of 1.0 fs. A cutoff distance of 1.6 nm was set for short-range van der Waals interactions and real-space electrostatic interactions between atomic partial charges. The Particle-Mesh Ewald summation method with an interpolation order of 5 and a Fourier grid spacing of 0.16 nm was employed to handle long-range electrostatic interactions in reciprocal space.

All tetraalkylphosphonium [BMB] IL systems were first energetically minimized using a steepest-descent algorithm and thereafter annealed gradually from 800 to 323 K within 20 ns. All annealed simulation systems were equilibrated in NPT (isothermal-isobaric) ensemble for 60 ns of physical time, maintained using Nosé-Hoover chain thermostat and Parrinello-Rahman barostat with time coupling constants of 0.5 and 0.2 ps, respectively, to control temperature at 323 K and pressure at 1 atm. Atomistic simulations of all simulation systems were further performed in NVT ensemble for 100 ns, and simulation trajectories were recorded at an interval of 100 fs for further microstructural analysis. Additional atomistic simulations were performed at a wide temperature range to address the dependence of the liquid densities of all studied tetraalkylphosphonium [BMB] ILs on temperature for comparative purposes.

### 3.2. Liquid Densities

**Figure 11:**
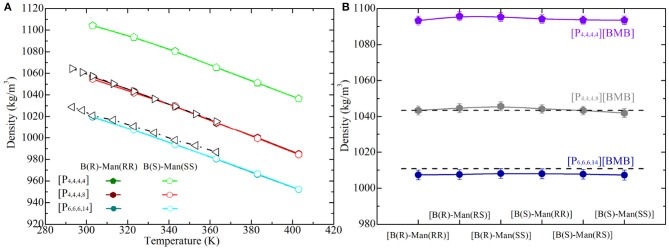
Liquid densities of **(A)** representative tetraalkylphosphonium [BMB] ILs at different temperatures and **(B)** all studied tetraalkylphosphonium [BMB] ILs at 323 K. Experimental data (dashed lines) for [P_4,4,4,8_][BMB] and [P_6,6,6,14_][BMB] ILs are derived from Shah et al. (2011) for comparison.

The liquid densities of all of the studied tetraalkylphosphonium [BMB] IL systems were calculated from current atomistic simulations at different temperatures ranging from 293 to 373 K with an interval of 20 K, and representative computational results are shown in Figure 11. In addition, experimental density data of [P_4,4,4,8_][BMB] and [P_6,6,6,14_][BMB] ILs taken from Shah et al. (2011) are also provided in Figure 11 for comparative proposes. The liquid densities of all tetraalkylphosphonium [BMB] ILs exhibit linear variations as temperature changes within the investigated temperature range. For a given [BMB] conformer, both the experimental and computational density results of tetraalkylphosphonium [BMB] ILs decrease with increasing number of carbon atoms in cations at specific temperatures, following that order [P_4,4,4,4_] > [P_4,4,4,8_] > [P_6,6,6,14_]. This is attributed to the reduced interactions between large tetraalkylphosphonium cations and [BMB] anions leading to their less efficient packing in heterogeneous IL matrices (Wang et al., 2014, 2017b). In addition, a good agreement is observed between experimental data and computational results for [P_4,4,4,8_][BMB] and [P_6,6,6,14_][BMB] ILs at all studied temperatures.

However, the molecular chiralities of [BMB] anions have a negligible effect on the liquid densities of these tetraalkylphosphonium [BMB] ILs, which may be attributable to elaborate interactions among all constituent ions in liquid environments. It is known that liquid density is a thermodynamic manifestation of all possible ion pair and ion cluster structures in local liquid environments. Even though [BMB] conformers have their intrinsic molecular volumes, as determined from DFT calculations, these [BMB] anions have delicate coordinations with tetraalkylphosphonium cations in heterogeneous IL matrices, leading to their comparable liquid densities as shown in Figure 11B. In addition, the agreement between experimental data and simulation results is remarkably good, with a maximum deviation of approximately 0.3% and 0.5% for [P_4,4,4,8_][BMB] and [P_6,6,6,14_][BMB] ILs, respectively. These computational data suggest that there might be multiple [BMB] conformers in experimental tetraalkylphosphonium [BMB] IL samples depending on the relative ratio of R- and S-mandelic acids in the reaction vessel. Therefore, an advanced synthesis method and separation technology would be useful for better extraction of varied [BMB] conformers from IL samples due to their specific coordinations with cation groups like alkali metal ions.

### 3.3. Hydrogen Bonding Interactions

HB interactions between ion species in ILs are one of the most important interactions and have a significant effect on ion packing structures in local ionic environments (Hunt et al., 2015; Matthews et al., 2015; Sha et al., 2016; Wang et al., 2017a; Wang, 2018; Pei and Laaksonen, 2019; Wang et al., submitted). In the current work, we studied HB interactions between tetraalkylphosphonium cations and [BMB] anions with varied molecular chiralities. For all these tetraalkylphosphonium [BMB] ILs, HP atoms in cations (as labeled in Figure 1) are potential HB donor sites (Wang et al., 2017b; Wang et al., submitted), and all three oxygen atom types in anions (as labeled in Figure 1) are preferential HB acceptors.

**Figure 12:**
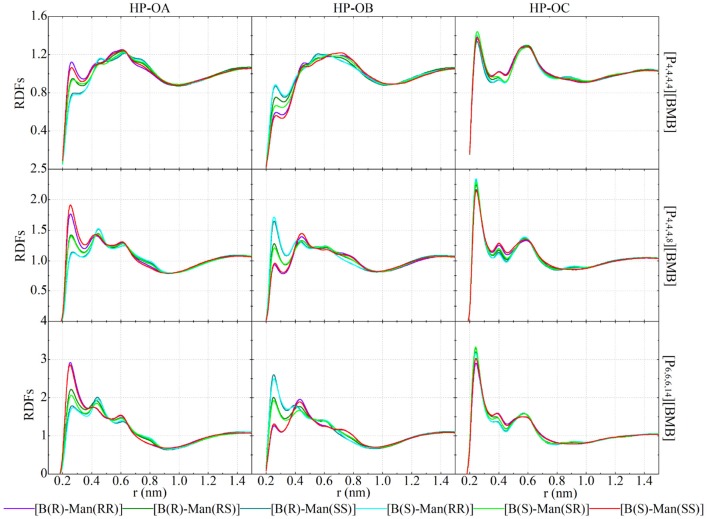
RDFs of HP atoms in [P_4,4,4,4_] **(upper panel)**, [P_4,4,4,8_] **(middle panel)**, and [P_6,6,6,14_] **(lower panel)** cations with three oxygen atom types (OA, OB, and OC atoms as labeled in Figure 1) in [BMB] anions with varied molecular chiralities.

Figure 12 presents representative HP-O radial distribution functions (RDFs) for HB coordinations of tetraalkylphosphonium cations with [BMB] anions. For given tetraalkylphosphonium cations, the RDF peak intensities of HP atoms in coordinating OA and OB atoms in [BMB] anions follow the three categories of [BMB] conformers as discussed in the previous section. The V-shaped B(R)-Man(RR) and B(S)-Man(SS) [BMB] conformers have strong HB associations, the bent B(R)-Man(RS) and B(S)-Man(SR) [BMB] conformers have intermediate HB interactions, and the twisted B(R)-Man(SS) and B(S)-Man(RR) [BMB] conformers have weak HB coordinations with HP atoms in tetraalkylphosphonium cations via OA atoms (left column in Figure 12). However, an opposite effect is observed for HP atoms in HB interactions with OB atoms in [BMB] anions, as clearly manifested in the middle column of Figure 12. In addition, OC atoms in all [BMB] conformers have stronger interactions with HP atoms in tetraalkylphosphonium cations than OA and OB atoms, irrespective of the molecular chiralities of [BMB] conformers, as shown in the right column of Figure 12. These computational results indicate that OA and OB have competitive HB interactions with HP atoms in tetraalkylphosphonium cations and that their HB capabilities depend on the molecular chiralities, conformational flexibilities, and steric hinderance effects of [BMB] conformers. The V-shaped B(R)-Man(RR) and B(S)-Man(SS) [BMB] conformers have small cavities in phenyl-oxalato-phenyl domains, which prevent central polar groups of tetraalkylphosphonium cations from approaching, and thus OB atoms in local cavities have weak HP-OB interactions. The HB acceptor sites outside cavities, such as OC and OA atoms, have preferential HB interactions with HP atoms in tetraalkylphosphonium cations. For bent B(R)-Man(RS) and B(S)-Man(SR) [BMB] conformers, both two OA and two OB atoms have one site inside and one outside local cavities, and thus OA and OB atoms have comparable HB associations with neighboring tetraalkylphosphonium cations via HP atoms. The OA and OB atoms have opposite distributions in twisted B(R)-Man(SS) and B(S)-Man(RR) [BMB] conformers to in V-shaped B(R)-Man(RR) and B(S)-Man(SS) [BMB] conformers, and thus they have distinct HB coordinations with tetraalkylphosphonium cations via HP atoms.

Enlarging tetraalkylphosphonium cation sizes from [P_4,4,4,4_] (upper panels in Figure 12) to [P_4,4,4,8_] (middle panels in Figure 12) and [P_6,6,6,14_] (lower panels in Figure 12) leads to enhanced HB interactions of HP in cations with all three oxygen atom types in [BMB] anions. This observation is explained by enhanced segregation of polar groups consisting of central oxalato moieties in anions and central P(CH_2_)_4_ groups in cations in apolar networks consisting of phenyl groups in anions and remaining alkyl moieties in cations with a gradual addition of apolar alkyl groups to tetraalkylphosphonium cations, as has been intensively discussed in previous works (Wang et al., 2017b; Wang, 2018).

### 3.4. Microscopic Liquid Structures

**Figure 13:**
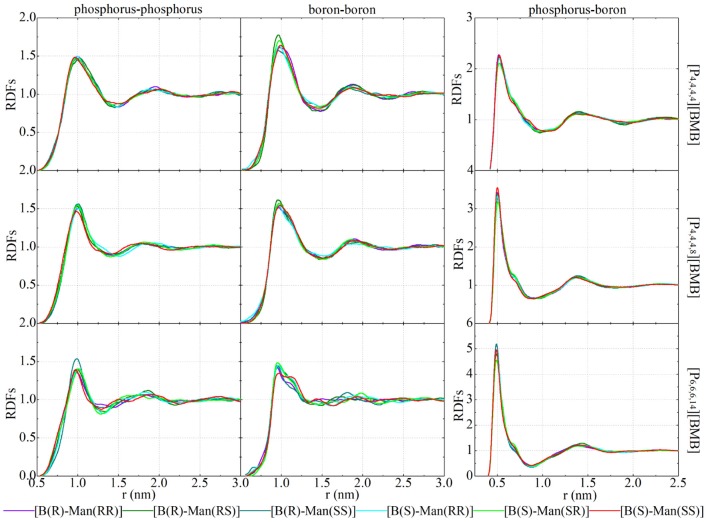
RDFs between phosphorus atoms in cations and boron atom in anions in [P_4,4,4,4_][BMB] **(upper panel)**, [P_4,4,4,8_][BMB] **(middle panel)**, and [P_6,6,6,14_][BMB] **(lower panel)** ILs with anions of varied molecular chiralities.

Figure 13 esentative site-site RDFs of cation-cation, anion-anion, and cation-anion pairs for all of the studied tetraalkylphosphonium [BMB] ILs. The central phosphorus atoms in tetraalkylphosphonium cations and boron atoms in [BMB] anions are taken as reference sites to calculate these RDFs, which are useful for depicting microscopic liquid structures between ion species in tetraalkylphosphonium [BMB] ILs. Both phosphorus-phosphorus and boron-boron RDF plots present very broad peaks at around 1.0 nm, with similar peak intensities in all of the studied tetraalkylphosphonium [BMB] ILs. The molecular chiralities of [BMB] anions have minimal effect on the spatial distributions of boron atoms in [BMB] anions around phosphorus atoms in tetraalkylphosphonium cations, and *vice versa*. These computational data are explained by strong Coulombic interactions among phosphorus and boron atoms in tetraalkylphosphonium cations and [BMB] anions, respectively. In addition, Coulombic interactions between central polar groups in tetraalkylphosphonium cations and [BMB] anions are overwhelmingly stronger than preferential intermolecular HB interactions, the latter of which have difficulty mediating the distributions of anions around cations but do fine-tune the local orientations of [BMB] conformers to maximize their HB interactions with tetraalkylphosphonium cations in local ionic environments.

Enlarging molecular sizes of tetraalkylphosphonium cations from [P_4,4,4,4_] (upper panels in Figure 13) to [P_4,4,4,8_] (middle panels in Figure 13) and [P_6,6,6,14_] (lower panels in Figure 13) leads to increased intermolecular interactions between phosphorus and boron atoms in respective ion groups, as clearly manifested in the right column of Figure 13, owing to enhanced segregation of polar groups in apolar networks in heterogeneous IL matrices (Wang et al., 2017b; Wang, 2018).

In addition, we calculated the total X-ray scattering structural function, *S*(*q*), using a summation of atom type-based partial components as $S\left(q\right)=\overset{n}{\underset{i=1}{\sum }}\overset{n}{\underset{j=1}{\sum }}S_{ij}\left(q\right)$, to explore the overall effect of the molecular chiralities of [BMB] anions and variations of tetraalkylphosphonium cation structures on microstructural ordering characteristics in tetraalkylphosphonium [BMB] ILs. *S*_*ij*_(*q*) is a partial structural function between atom types *i* and *j* and is given by

$$\begin{array}{r} S_{ij}\left(q\right)=\frac{\rho_{0}x_{i}x_{j}f_{i}\left(q\right)f_{j}\left(q\right)\int_{0}^{L/2}4\pi r^{2}\left[g_{ij}\left(r\right)-1\right]\frac{sin\left(qr\right)}{qr}W\left(r\right)dr}{{\left[\sum_{i=1}^{n}x_{i}f_{i}\left(q\right)\right]}^{2}}. \end{array}$$

*x*_*i*_ and *x*_*j*_ are mole fractions, and *f*_*i*_(*q*) and *f*_*j*_(*q*) are X-ray atomic form factors of atom types *i* and *j* in simulation systems (Prince and Wilson, 2004), respectively. *g*_*ij*_(*r*) is the partial RDF between atom types *i* and *j*, including both intra- and intermolecular pairs. $\rho_{0}=\frac{N_{atom}}{<L^{3}>}$ refers to an averaged atom number density of a simulation system, and *L* is the simulation box length. *W*(*r*) is a Lorch window function defined as $W\left(r\right)=\frac{sin\left(2\pi r/L\right)}{2\pi r/L}$, which is used to minimize the effect of finite truncation of *r* for the calculation of *g*_*ij*_(*r*). For a simulation system of moderate size, this window function does not alter the physical meaning of peaks and anti-peaks in *S*(*q*) plots (Annapureddy et al., 2010; Hettige et al., 2012; Wu et al., 2016; Wang et al., 2017b).

**Figure 14:**
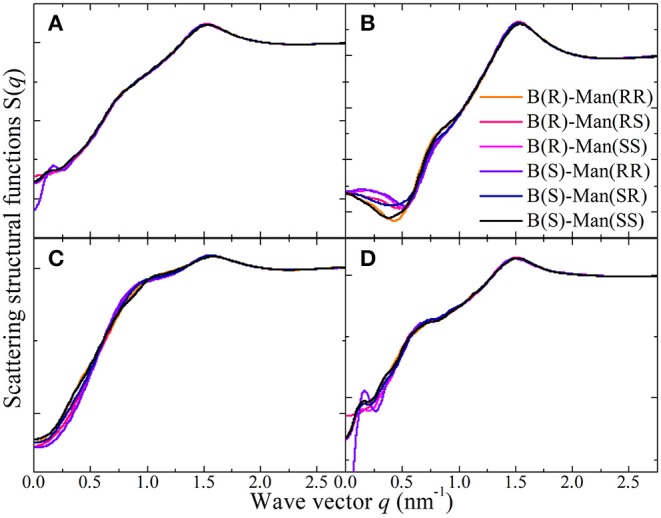
**(A)** The total X-ray scattering structural functions *S*(*q*) in the range of *q* ≤ 2.75 nm^−1^ for [P_4,4,4,4_][BMB] IL at 323 K with anions of varied molecular chiralities. These total X-ray scattering structural functions are further partitioned into partial structural functions for **(B)** cation-anion/anion-cation, **(C)** cation-cation, and **(D)** anion-anion subcomponents.

Figure 14A presents the total X-ray scattering structural functions *S*(*q*) for [P_4,4,4,4_][BMB] ILs in the range of *q* ≤ 2.75 nm^−1^, which is the most relevant region and is associated with intermolecular correlations between tetraalkylphosphonium cations and [BMB] anions in heterogeneous IL matrices. Features at *q* values larger than 2.75 nm^−1^ are mostly intramolecular in nature, which are fairly easy to assign and thus are not discussed in the current work. Two prominent peaks located at ~0.76 nm^−1^ and ~1.51 nm^−1^ are shown in these total X-ray scattering structural functions for [P_4,4,4,4_][BMB] ILs with anions of varied molecular chiralities. Such a two-peak-plot is a general feature for tetraalkylphosphonium ILs and is mainly attributed to the bulky and voluminous structures of cations and their strong coordinations with anions via strong Coulombic interactions between polar groups in the respective ion species and preferential hydrophobic interactions among alkyl and phenyl moieties in the constituent ions (Amith et al., 2016; Hettige et al., 2016; Wang et al., 2017b; Wang et al., submitted). These peak positions are essential characteristic hallmarks of their microstructural landscapes, indicating particular microscopic ion ordering phenomena at different length scales in tetraalkylphosphonium [BMB] IL matrices (Annapureddy et al., 2010; Hettige et al., 2012; Amith et al., 2016; Wu et al., 2016; Wang et al., 2017b; Wang, 2018; Wang et al., submitted).

The peaks in the intermediate *q* range around 0.76 nm^−1^ are indicative of a mesoscopic liquid organization characterized by positive-negative charge alternations in IL matrices (Canongia Lopes and Pádua, 2006; Annapureddy et al., 2010; Amith et al., 2016; Wu et al., 2016; Wang et al., 2017b; Weyman et al., 2018; Wang et al., submitted). This charge ordering behavior results from the need to maintain a lattice-like arrangement of cations and anions to minimize Coulombic interactions in IL matrices and is thus associated with a length scale between ions of the same charge separated by ions of opposite charge (Annapureddy et al., 2010; Hettige et al., 2012; Wu et al., 2016; Wang et al., 2017b; Wang, 2018; Weyman et al., 2018; Wang et al., submitted). For some ILs, this charge alternation peak is present as a weak shoulder because of an almost complete cancellation of peaks and anti-peaks, which offsets this ordering phenomena at intermediate length scale (Amith et al., 2016; Wang et al., 2017b). The prominent peaks at high *q* values near 1.51 nm^−1^ are associated with short-range adjacency correlations originating from the nearest neighboring ion species (Annapureddy et al., 2010; Hettige et al., 2012, 2016; Wu et al., 2016; Wang et al., 2017b; Wang, 2018; Wang et al., submitted). In fact, this peak mainly originates from apolar adjacency correlations between ion species, as has been verified in previous studies (Hettige et al., 2012; Amith et al., 2016; Wu et al., 2016; Wang et al., 2017b; Weyman et al., 2018).

Either for the total X-ray scattering structural functions or the partial structural functions for cation-anion/anion-cation (Figure 14B), cation-cation (Figure 14C), and anion-anion (Figure 14D) subcomponents in [P_4,4,4,4_][BMB] ILs, the dependence of charge alternations and adjacency correlations in these scattering structural functions on the molecular chiralities of [BMB] anions is negligible. This is expected, as scattering structural functions are intrinsically determined by RDFs between different atom pairs. It is shown in Figure 13 that the molecular chiralities of [BMB] anions have minimal effects on phosphorus-phosphorus, phosphorus-boron, and boron-boron RDFs, which leads to comparable scattering structural functions for [P_4,4,4,4_][BMB] ILs with anions of varied molecular chiralities. Therefore, it can be asserted that [P_4,4,4,4_][BMB] ILs are characterized by similar liquid morphologies, which are independent of the molecular chiralities of [BMB] anions. Additional computational results indicating that the molecular chiralities of [BMB] anions have minimal influence on the total and partial scattering structural functions were also obtained for [P_4,4,4,8_][BMB] and [P_6,6,6,14_][BMB] ILs.

**Figure 15:**
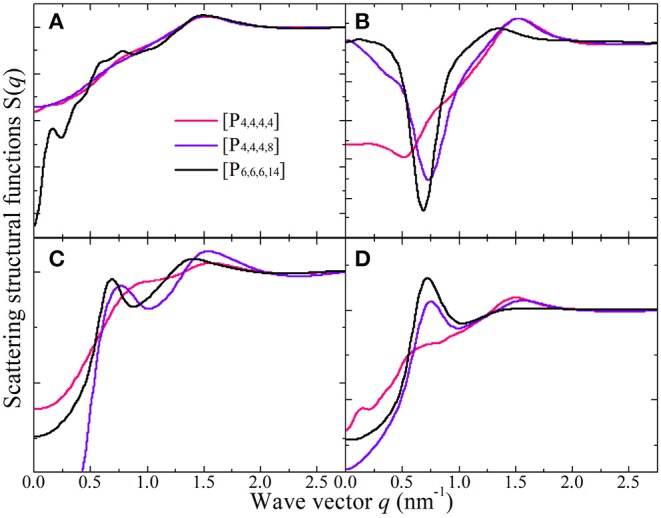
**(A)** The total X-ray scattering structural functions *S*(*q*) in the range of *q* ≤ 2.75 nm^−1^ for tetraalkylphosphonium [BMB] ILs at 323 K with anions characterized by the B(R)-Man(SS) configuration. These total X-ray scattering structural functions are further partitioned into partial structural functions for **(B)** cation-anion/anion-cation, **(C)** cation-cation, and **(D)** anion-anion subcomponents.

Figure 15 presents representative total and partial X-ray scattering structural functions for tetraalkylphosphonium [BMB] ILs at 323 K with anions characterized by the B(R)-Man(SS) configuration. In the intermediate *q* range, both cation-cation (Figure 15C) and anion-anion (Figure 15D) subcomponents contribute to positive intensities, and the cross-term cation-anion/anion-cation subcomponent (Figure 15B) exhibits prominent anti-peaks in the same *q* range. A combination of these peaks and anti-peaks contributes to prominent peaks registered in the intermediate *q* range in the total X-ray scattering structural functions shown in Figure 15A. Independent of tetraalkylphosphonium [BMB] ILs, the charge alternation peaks always appear as a result of the cancellation of prominent positive contributions from same-charge subcomponents and distinct negative-going peaks from cross-term contributions (Annapureddy et al., 2010; Amith et al., 2016; Wu et al., 2016; Wang et al., 2017b; Wang, 2018; Weyman et al., 2018). This observation indicates that, at specific locations where one expects to find a same-charge ion, there is a systematic absence of ions with opposite charge (Annapureddy et al., 2010; Hettige et al., 2012, 2016; Amith et al., 2016; Wu et al., 2016; Wang et al., 2017b; Wang, 2018; Wang et al., submitted). In addition, all partial scattering structural functions present positive contributions to adjacency correlations between neighboring ion species at high *q* values around 1.51 nm^−1^ but with different portions of contribution.

**Figure 16:**
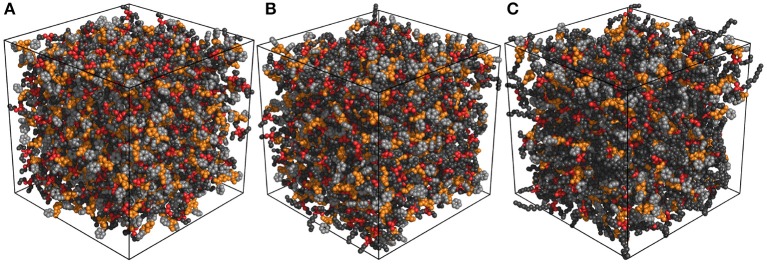
Representative liquid morphologies of **(A)** [P_4,4,4,4_][BMB], **(B)** [P_4,4,4,8_][BMB], and **(C)** [P_6,6,6,14_][BMB] ILs with anions characterized by the B(R)-Man(SS) configuration. Polar domains consist of oxalato moieties (orange) in [BMB] anions and central P(CH_2_)_4_ groups (red) in tetraalkylphosphonium cations, and apolar entities consist of phenyl units (light gray) in [BMB] anions and the remaining alkyl groups (dark gray) in tetraalkylphosphonium cations.

Different to the effect of the molecular chiralities of [BMB] anions on the scattering structural functions for tetraalkylphosphonium [BMB] ILs, the variation of cation structures from [P_4,4,4,4_] to [P_4,4,4,8_] and [P_6,6,6,14_] has a significant effect on RDFs (Figure 13), scattering structural functions (Figure 15), and microscopic liquid morphologies (Figure 16). Enlarging cation sizes from [P_4,4,4,8_] to [P_6,6,6,14_] leads to a concomitant shift of scattering peaks for charge alternations and adjacency correlations to lower *q* values (corresponding to larger characteristic distances for apolar domains in real space) in total and partial scattering structural functions (Amith et al., 2016; Wu et al., 2016; Wang et al., 2017b; Wang, 2018; Wang et al., submitted). These computational results are explained by dispersed distributions of polar domains consisting of central oxalato moieties in anions and central P(CH_2_)_4_ groups in cations in apolar networks consisting of phenyl groups in anions and remaining alkyl moieties in cations and also the expansion of apolar networks in IL matrices with lengthening alkyl chains in tetraalkylphosphonium cations, as shown in Figure 16. Such a microstructural change in tetraalkylphosphonium [BMB] IL matrices has an impact on the number of counterions present in the solvation shells of a given ion and an even bigger effect on the distributions of (same-charge) ions nearby and thus contributes to distinct changes in peak positions for charge alternations and adjacency correlations at low and high *q* values, respectively. This observation is, in general, consistent with computational results for imidazolium oxalatoborate ILs with cations with varied alkyl chains (Wang et al., 2017a; Wang, 2018) and for ILs consisting of tetraalkylphosphonium cations coupled with chloride, bromide, dicyanamide, and bis(trifluoromethylsulfonyl)imide anions (Hettige et al., 2012, 2016; Wang et al., 2017b; Wang et al., submitted).

## 4. Concluding Remarks

Spiroborate anion-based inorganic electrolytes and ILs have fascinating physicochemical and structural properties and have promising applications in tribology and electrochemistry. Besides variations in cation structures, the molecular chiralities of spiroborate anions have a significant effect on their macroscopic functionalities in these applications due to their specific binding structures and preferential interactions with neighboring ions in liquid matrices. A thorough understanding of the delicate associations between spiroborate anions and the paired cation species will provide valuable information for the selection and design of suitable lubricants and solvent electrolytes with desirable physicochemical properties for their specific applications.

In the current study, we performed intensive DFT calculations to study the specific binding structures of [BMB] anions with varied molecular chiralities with representative alkali metal ions and the representative electronic properties of the alkali metal ion-[BMB] ion pair complexes. The optimized [BMB] conformers are characterized by V-shaped, bent, and twisted anion structures with varied electrostatic potential contours, conformational energies, and distinct alkali metal ion-[BMB] binding structures. Alkali metal ions have considerable associations with phenyl groups in V-shaped [BMB] conformers owing to preferential cation-π interactions.

In addition, we carried out extensive atomistic interactions to explore the effects of the molecular chiralities of [BMB] anions on the thermodynamics and microstructural properties of bulk tetraalkylphosphonium [BMB] ILs. It was revealed that oxygen atoms in [BMB] anions have competitive hydrogen bonding interactions with hydrogen atoms in tetraalkylphosphonium cations depending on the molecular chiralities of [BMB] anions and the steric hindrance effects of phenyl groups in [BMB] anions. However, the molecular chiralities of [BMB] anions have little effect on the liquid densities of tetraalkylphosphonium [BMB] ILs and the spatial distributions of boron atoms in anions around phosphorous atoms in cations. Enlarging tetraalkylphosphonium cation sizes from [P_4,4,4,4_] to [P_4,4,4,8_] and [P_6,6,6,14_] leads to enhanced cation-anion intermolecular hydrogen bonding and Coulombic interactions, as well as to distinct microscopic liquid structures as characterized by computational X-ray scattering structural functions, due to enhanced segregation of polar groups (consisting of central oxalato moieties in [BMB] anions and central P(CH_2_)_4_ groups in tetraalkylphosphonium cations) in apolar networks (composed of phenyl groups in [BMB] anions and the remaining alkyl moieties in tetraalkylphosphonium cations) in heterogeneous IL matrices.

## Data Availability Statement

The raw data supporting the conclusions of this article will be made available by the authors, without undue reservation, to any qualified researcher.

## Author Contributions

Y-LW designed DFT and atomistic simulations and performed DFT calculations. H-WP performed atomistic simulations. All authors wrote, reviewed, and edited all versions of this article.

### Conflict of Interest

The authors declare that the research was conducted in the absence of any commercial or financial relationships that could be construed as a potential conflict of interest.
